# Focal Point of Fanconi Anemia Signaling

**DOI:** 10.3390/ijms222312976

**Published:** 2021-11-30

**Authors:** Sudong Zhan, Jolene Siu, Zhanwei Wang, Herbert Yu, Tedros Bezabeh, Youping Deng, Wei Du, Peiwen Fei

**Affiliations:** 1 University of Hawaii Cancer Center, University of Hawaii, Honolulu, HI 96813, USA; SZhan@cc.hawaii.edu (S.Z.); ZWang@cc.hawaii.edu (Z.W.); HYu@cc.hawaii.edu (H.Y.); 2 Student Research Experience Program of University of Hawaii, Honolulu, HI 96822, USA; Jolenems@hawaii.edu; 3 Department of Chemistry, University of Guam, Mangilao, GU 96923, USA; bezabeht@triton.uog.edu; 4 Department of Quantitative Health Sciences, John A. Burns School of Medicine, University of Hawaii, Honolulu, HI 96813, USA; dengy@hawaii.edu; 5 Division of Hematology and Oncology, University of Pittsburgh School of Medicine, UPMC Hillman Cancer Center, Pittsburgh, PA 15232, USA; duw@upmc.edu

**Keywords:** DNA damage response, genome stability, genetic disease, fanconi anemia

## Abstract

Among human genetic diseases, Fanconi Anemia (FA) tops all with its largest number of health complications in nearly all human organ systems, suggesting the significant roles played by FA genes in the maintenance of human health. With the accumulated research on FA, the encoded protein products by FA genes have been building up to the biggest cell defense signaling network, composed of not only 22+ FA proteins but also ATM, ATR, and many other non-FA proteins. The FA D2 group protein (FANCD2) and its paralog form the focal point of FA signaling to converge the effects of its upstream players in response to a variety of cellular insults and simultaneously with downstream players to protect humans from contracting diseases, including aging and cancer. In this review, we update and discuss how the FA signaling crucially eases cellular stresses through understanding its focal point.

## 1. Introduction

Fanconi anemia (FA) is a rare human genetic disorder with an incidence rate of about one in 136,000 births, and half of FA patients are diagnosed prior to age 10 [1,2,3,4,5]. FA occurs equally in males and females and is found in all ethnic groups, but is more common among people of Ashkenazi Jewish descent, the Roma population of Spain, and black South Africans [3,4]. It is usually inherited as an autosomal recessive genetic disorder, but X-linked or autosomal-dominant inheritance has also been reported. FA is often associated with a progressive deficiency of all blood cells—red blood cells, white blood cells, and platelets. Affected individuals have an increased risk of developing a malignancy in blood-forming cells, such as acute myeloid leukemia (AML) or cancer of the head, neck, skin, gynecologic, or gastrointestinal systems [1,2,3,4,6,7,8].

It is unfortunate to say that health complications of FA have evidently indicated the functional significance of FA or FA-related genes. To date, the fact that FA patients who live into adulthood are likely to develop AML or a variety of solid tumors, has led to an exciting field of cancer research. Under the circumstance, understanding these FA or non-FA gene functions has become a new direction to advance cancer research. Indeed, many studies including ours have demonstrated numerous discoveries in advancing our understanding of cancer prevention, etiology, and treatment. Noting many published reviews on the relation between FA signaling and human diseases [1,2,3,4,5,6,9,10], we herein highlight how the FA proteins, located at the center of FA signaling, orchestrate all players involved in this signaling network to advance our understanding of cancer development and treatment. 

## 2. The Center or Focal Point of FA Signaling 

**Figure 1:**
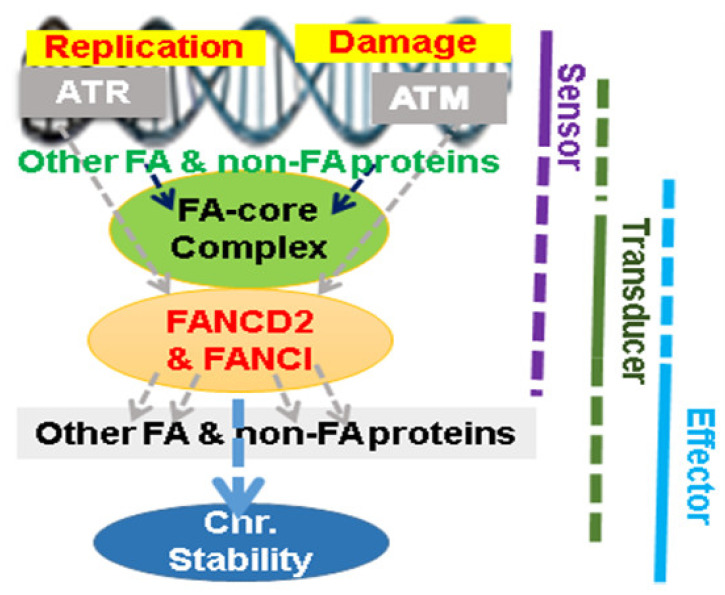
Schematic Representation of Focal Point of the FA Signaling Network. FA signaling is centered by FA group D2 protein (FANCD2) and its paralog (FANCI), which form the focal point of FA signaling. Both upstream and downstream of this focal point contain an undefined number of players. The focal point converges the effects of upstream players in response to a variety of cellular insults and works simultaneously with downstream players to guard genome stability and to ultimately prevent humans from contracting a variety of diseases, including aging and cancer. Further, each player of DDR/FA signaling (1) is working relatively in a phase of DNA damage responses. For example, FANCD2 and FANCI may mainly act as transducers; however, they can also be sensors or effectors as the dotted bold lines indicate at the right.

FA signaling is defined upon similar clinical, cellular, and molecular abnormalities displayed from at least 22 groups of FA patients. FANCD2 and its paralog FANCI stand at the center and become the focal point of this huge signaling network (Figure 1). The monoubiquitination of FANCD2 and FANCI is a critical event for the activation of FA signaling during DNA replication or upon DNA damage [2,9]. The FA proteins (FANCA, B, C, E, F, G, L, M, T, and possibly I) along with FA-associated proteins (FAAPs: FAAP 20/24/100 and MHF1/2, etc.) form the FA core complex to assure the activity of ubiquitin E3 ligase, which monoubiquitinates FANCD2 and its paralog/FANCI. Monoubiquitinated FANCD2 and FANCI, namely, ID complex, in turn, work with the downstream players, including the rest of the FA proteins and other non-FA proteins to repair DNA damage through mechanisms of BER, NER, TLS, HR, NHEJ [1], and/or possibly other cellular processes, such as splicing [11], to maintain genome stability. Therefore, the monoubiquitination/activation of FANCD2-FANCI appears to be pivotal in holding/guarding the normal functions of the upstream and downstream players of FA signaling, which also include many other proteins yet to be recognized (Figure 1). Due to the indefinite nature of upstream and downstream FA signaling, studying the functions of the center players appears to be more rational than any others in understanding how FA signaling prevents normal human cells from going awry for neoplastic transformation.

The FA complementation groups currently include FANCA (also called FANCH), FANCB, FANCC, FANCD1 (also called BRCA2), FANCD2, FANCE, FANCF, FANCG (also called XRCC9), FANCI, FANCJ (also called BRIP1 or BACH1), FANCL, FANCM and FANCN (also called PALB2), FANCO (also called Rad51C), FANCP (also called SLX4 or BTBD12), FANCQ (also called ERCC4 or XFP), FANCR (also called Ra51), FANCS (also called BRCA1), FANCT (also called UBE2T), FANCU (XRCC2), FANCV (also called REV7 or MAD2L2), and FANCW (also called RFWD3) [1,2]. The members of the FA proteins do not share sequence similarity, but they are related by their assembly into shared nuclear protein complexes to constitute a common cell defense signaling network (Figure 1). The monoubiquitination of FANCD2 and FANCI proteins lies at the heart of this signaling network, which triggers the recruitment of DNA repair factors. A major roadblock in our understanding of this fundamental cell defense mechanism arises from the challenge with fully understanding monoubiquitinated FANCD2 and FANCI proteins.

FANCD2 (and FANCI) monoubiquitination leads to the retention of the ID complex at sites of stalled replication or the damaged DNA. FA cells are exquisitely sensitive to agents that increase stalled replication or DNA damage, such as UV, platinum, or mitomycin C (MMC) [12,13,14,15]. Mutations in the genes that encode proteins in the FA core complex, the ID complex, or some subcomplexes are present in over 97% of FA cases [1,16]. The FA core complex acts as the ubiquitin ligase E3 to monoubiquitinate the FANCI–FANCD2 at the site of DNA damage or stalled replication forks [14,17]. It is thought that this recruits downstream nucleases containing ubiquitin-binding domains to repair the damaged DNA through a variety of repair mechanisms aforementioned [15,18]. USP1 acts with UAF1 during DNA replication or repair processes to remove the monoubiquitin of FANCD2–FANCI and turn off the process, which allows a highly choreographed monoubiquitination and deubiquitination to occur at the focal point of the FA signaling. Then, we relatively organized the passages under FANCD2, FANCI, and FANCD2 and FANCI for improved readability, although they function very closely which is fairly undividable over the physiological point of view. 

### 2.1. FANCD2

FANCD2 is monoubiquitinated in response to DNA damage, resulting in its localization to nuclear foci with FANCS, FANCD1, and others involved in homology-directed DNA repair, nonhomologous end joining, postreplication repair, etc. Accumulated studies indicate that FANCD2 acts in coordination with many repair proteins known and/or yet to be identified in nearly all phases of the DNA damage responses, sensing, signal transduction, and execution of repair (Figure 1). As such, the phosphorylation of FANCD2 at Ser222, initiated by ATM, contributes to arresting cells in the S phase of a cell cycle [19], which may be counted in the sensor phase, but it can be a typical transducer in terms of being a focal player in the FA signaling network or an effector in terms of its involvement in a specific type of DNA repair. From the prospective of checkpoint mechanisms centering on the coordinated events [20], the DNA damage repair function of FANCD2 is equally crucial in arresting or resuming cell proliferation or in helping eliminate the overdamaged cells. Under such situations, FANCD2 acts possibly as a messenger. To date, FA or FA-associated proteins not only perform the common role in signaling but also conduct tasks in a pathway-independent manner [1,2,21,22,23,24]. Huge attention has been given to the DNA damage condition of cells where monoubiquitination/activation of FANCD2 takes place for repairing damaged DNA. In contrast, little attention has been given to FANCD2′s roles in a particular phase of a cell cycle without an exposure of DNA damage agents. FANCD2 is not constitutively monoubiquitinated through all phases of a cell cycle, rather monoubiquitinated/activated FANCD2 is only present in the S phase of a cell cycle [25]. This basal level of FANCD2 monoubiquitination occurring in normally growing cells has been demonstrated to be essential for replication origins to fire at a normal rate [26]. Conversely, the loss of the basal level of FANCD2 monoubiquitination leads to a slow rate of replication origin firing. Compared to studies on FANCD2′s involvement in the S phase, how FANCD2 plays specific roles in the M phase of a cell cycle and appears to underperformed, and these studies will certainly better our understanding of tumor cell division. 

FA cells have high incidences of aneuploidy and micronucleation, often occurring as a result of chromosome missegregation [27]. In addition to chromosomal instability resulting from defected FA signaling [28], genetic models inhibiting FANCD2 monoubiquitination have also demonstrated deregulated cell proliferation/growth [29], consistent with the reported findings of a dysregulated cell cycle in FA cells [30]. Following the impairment of FA signaling or inactivated FANCD2, the mechanistic consequences extend past deregulation in DNA damage responses (DDR) and aberrant replications. FANCD2/FA signaling may play emerging roles in the M phase of a cell cycle [31,32,33,34,35], such as how the CDK-mediated phosphorylation of FANCD2 promotes mitotic entry [36]. In addition, its roles may contain other cellular processes under nonstressed conditions. For instance, FANCD2 is involved in maintaining the stability of common fragile sites [37], and its phosphorylation may inhibit the function of the ID complex and the function of FA signaling in the absence of DNA damage [38].

While fathoming the functional varieties of FANCD2, we started to characterize an unrecognized form of FANCD2, namely, FANCD2-V2 in contrast to the long-known one, FANCD2-V1 [39]. This was done by sequence similarity searching from NCBI. We found a 60 bp longer version of FANCD2 (V2) coding cDNA, encoding a protein carrying more than 95% of homology with the known “FANCD2 (V1)” protein. As this distinction was not previously described, many of the reported functions of FANCD2 could reflect the properties of either V1, V2, or both. Importantly, FANCD2-V2 exhibits greater association with nonmalignant cells compared to malignant cells; conversely, FANCD2-V1 is expressed more in malignant cells. The different expression patterns of FANCD2-V1 and V2 thereby emerge to be an important biological trait, possibly demarcating premalignancy from malignancy. To this point, how two forms of FANCD2 cooperate upon DNA damage, during DNA replication, or under non-S phases of a cell cycle are questions that are yet to be addressed. 

Our following studies also showed that the expression patterns of FANCD2-V1 and V2 are involved in the use of an alternative polyadenylation site (APS), which is regulated by DNA methylation in the distal or proximal regions of APS (Me-D and Me-P) [40]. The ratio of Me-D/Me-P was significantly higher (*p* < 0.01) in tumor samples than the matched normal tissues by analyzing publicly available datasets (*n* > 2500 in total, across seven types of human cancer). This discovery represents another important biologic variation that may be capable of marking nonmalignancy from malignancy. 

To better understand how Me-D/Me-P and V1/V2 derived from FA singling to be promising biomarkers, their biological bases need to be further determined. We reported that TRK-Fused Gene (TFG) was a specific functional partner for FANCD2-V2 in early cellular responses to DNA damage, but not for FANCD2-V1. As such, FANCD2-V2 forms nuclear foci upon DNA damage, and both its focus appearance and disappearance are earlier than FANCD2-V1 [41]. However, this was not shown in cells harboring mutated TFG compared to wtTFG-carrying cells. These functional studies unlock in-depth insights into maintaining genome stability performed by the FA signaling and further validate the translational capability of turning V1/V2 and Me-P/Me-D into effective biomarkers for preventing, diagnosing, and/or treating human cancers

### 2.2. FANCI

Among the listed 22 FA complementation groups, FANCI was discovered as a relatively new complementation group of FA, which acts as a paralog of FANCD2 required for DNA repair [42,43]. It seems to be shadowed under FANCD2, but its importance was soon displayed in the chicken DT40 cell system [44]. As reported, multiple alanine substitution mutations in six conserved and clustered Ser/Thr-Gln motifs of FANCI largely abrogate monoubiquitination and focus formation of both FANCI and FANCD2, resulting in a loss of DNA repair function. Therefore, FANCI phosphorylation may serve as a molecular switch in activation of the ID complex/FA signaling. In the meantime, how FANCI works in concert with FANCD2 has become the focus in the field of FA signaling research. The FANCI foci were found to be colocalized perfectly with the FANCD2 foci, which brought up the concept of the FANCI–FANCD2 complex (ID complex) or the center of FA signaling [45]. Apart from the important roles of FANCI in the ID complex in response to DNA damage or during DNA replication to couple with ATR [1,46], FANCI, like FANCD2, also possesses important cellular functions in the other phases of a cell cycle by directly integrating into other signaling pathways [24,47]. In particular, FANCI was recently found to also be a switch between repair and apoptosis. As known, FANCI heterodimerizes with FANCD2 to initiate the excision of interstrand crosslinks (ICLs) when ICL lesions occurred in DNA [1]. However, FANCI alternatively interacts with a proapoptotic factor, PIDD1, to enable PIDDosome (PIDD1-RAIDD-caspase-2) formation and apoptotic death [48]. Clearly, FANCI changes its partner from FANCD2 (repair) to PIDD1 (apoptosis) signaling, possibly under the circumstance of ICL repair failure. This was done specifically by removing endonucleases downstream of the ID complex to increase DNA damage or allow damaged cells into mitosis [48]. This study unveils decision making at the time of ICL occurring in a cell context-dependent manner in eukaryotes and suggests damaged cells can diverge from apoptosis when de novo lesion repair has succeeded, together providing in-depth insights into the sensitivity/resistance of ICL/DNA damage-related chemotherapeutic agents. 

The above functional highlights for FANCI demonstrate its extreme importance across a variety of cellular processes. This was further strengthened by numerous FANCI variations directly associated with many human cancers. Its germline mutations were found in AML [49] or related to the early onset of breast cancer [50], and its epigenetic change was directly involved in nasopharyngeal carcinoma [51], and others were involved in gastric cancers [52]. The functional importance of FANCI is also evidently shown in the conditional inactivation model for FANCI in mice [53]. Here, FANCI -/- mice displayed typical FA features such as developmental defects in utero or limb, microphtalmia, cell sensitivity to MMC, and a malfunctioned hematological system. The defective FANCI also leads to a strong meiotic phenotype and severe hypogonadism. At the molecular level, FANCI was consistent with a role in meiotic recombination and, unlike FANCD2, interacting with RAD51 and in stimulating a D-loop formation [53]. All of these display distinct functions of FANCI and its common functions shared with FANCD2, showing both ID-complex dependent and independent manners [53]. 

### 2.3. FANCD2 and FANCI

The FA signaling converges on its focal point (the ID complex), which is not only a substrate for the FA core complex but also a potential platform for recruiting downstream FA signaling players, including nucleases and other FA and non-FA proteins for DNA damage repair [15,54] (Figure 1). Significant advances have been made in the structural characterization of FA proteins. The crystal structure revealed several key phosphorylation sites in FANCI for its role in the function of the ID complex. In particular, the ATR-kinase substrate sites in FANCI, S555, T558, and T564 are exposed on a surface adjacent to the FANCD2 interface. In cells, the integrity of these sites is essential for FANCD2 monoubiquitination [15,44], and in vitro their phosphorylation or phosphomimic mutation leads to the stabilization of the ID complex on DNA [15]. FANCI forms a heterodimer with FANCD2 upon DNA damage and protects the deubiquitination of FANCD2 to better clamp DNA together. In addition to monoubiquitination and deubiquitination of FANCD2 and FANCI controlled by the FA core complex and USP1, respectively [18,20,21], this dynamic balance can also be regulated by a ribosomal protein S27-like which connects p53 signaling [55,56]. Ribosomal protein S27-like (RPS27L), a direct p53 target, plays an important role in the maintenance of genome integrity [57,58,59,60]. RPS27L was found to bind to FANCD2 and FANCI to prevent their degradation via the autophagy–lysosome system. Conversely, its inactivation impairs FA signaling by destabilization of the FANCD2 and FANCI/the ID complexes [55]. Here, RPS27L is an evolutionarily conserved ribosomal protein, distinct from the commonly known proteins for DDR. Therefore, it is another niche of studies questioning how many other known or unknown non-DDR proteins are involved in the regulation of FANCD2 and FANCI. 

Currently, the structure study provides much in-depth insight into protein–protein interactions, as such an interaction of FANCD2 and FANCI is attributed at least to armadillo (ARM) repeats and an EDGE motif at the C-terminus of FANCI [61]. A central step in the activation of the FA signaling is the monoubiquitination of the FANCD2 and FANCI proteins, which occurs within chromatin. DNA binding of FANCI–FANCD2 is required for monoubiquitination and activation of the FA signaling. Despite the numerous important findings, such as that FANCD2 binding to H4K20me2 is essential for repairing DNA crosslinks [62], how this activates DNA repair remains largely unclear. In addition, our studies on FANCD2 variants demonstrate an in-depth understanding of how FA signaling is timely in guarding genome stability, which may bring promising biomarkers that are useful for cancer prevention and/or earlier cancer diagnosis. Similarly, what are are the variants of FANCI like? To date, there is little known as to the roles of the FANCI variants. Further, the above functions of FANCD2 or FANCI discussed basically result from their nature as nuclear proteins involved in the ID complex for DNA damage repair. However, both FANCD2 and FANCI have been reported to play important roles beyond functioning within the ID complex. FANCD2 has been found to protect cells against ferroptosis-mediated damage in bone marrow stromal cells (BMSCs) [63]. It is also found to be required for the repression of germline transposable elements [64]. Similarly, FANCI has been found to play roles in ribosome biogenesis [65] and in cooperating with IMPDH2 during lung carcinogenesis [66]. We believe FANCD2 and FANCI will definitely perform many other functions in a manner of the ID-complex dependent or independent, which are yet to be studied. 

Germline FA gene mutations have been directly associated with many cancers including breast, ovarian, and pancreatic cancer owing to the defects relating to FANCD1/N/C and/or/G [67,68,69,70,71]. Specifically, mutations in FANCD1 (also called BRCA2) have an 82% lifetime risk of breast cancer, and a 23% risk of ovarian cancer [70,71]. These genetic studies support Dr. Swift’s prediction made about 50 years ago [72] that FA heterozygotes have an increased risk of cancer and provide further support to the concept that FA proteins play important roles as tumor suppressors. 

Somatic inactivation of the FA pathway could stem from the impairment of any FA proteins. Among these, the hypermethylated FANCF promoter, resulting in a compromised FA signaling, was found in 6.7% to 30% of tested tumor cell lines including testis, lung, ovarian, and cervical cancer lines [73,74,75]. Reduced levels of FANCA and FANCD2 in AML and breast cancer, respectively, have also been reported, although the causes of these reduced FANCA and FANCD2 levels remain unknown [76,77,78]. Therefore, the overall functional heterozygosity of the FA signaling may make a more significant contribution to the increased risk of human cancer, particularly as many more alterations occurring in genes are encoding proteins directly or indirectly affecting the functions of FA signaling. They also appear to occur more in tumor cells, compared to the genetic heterozygosity occurring only at the FA gene level. 

In our earlier studies, we discovered that a new form of FANCL protein, named FAVL, dysregulates the FA signaling in non-FA human tumor cells and acts as a tumor promoting factor by inactivating FANCD2 and FANCI/the ID complex [79,80,81,82]. We also found that the convergence of the FA and the human homolog of yeast Rad 6 (HHR6) pathways plays essential roles in maintaining genome stability and suppressing the development of human cancer [82,83,84,85,86]. Furthermore, we reported how inactivated FANCD2 gains a new role in promoting tumorigenicity, and how activated FANCD2 functions in the S phase of each normal cell cycle [26]. These studies have substantially improved our understanding of how functional heterozygosity of FA signaling promotes non-FA human tumorigenesis.

To date, the functional significance of germline variations in FA genes has been frequently reported [2,87]. For instance, patients with FA have an increased risk for head and neck squamous cell carcinoma (HNSCC). The authors sought to determine the prevalence of undiagnosed FA and FA carriers among patients with HNSCC, and they found FA gene variations in 44% of the patients tested [88]. In this study, an increased burden or mutation load of FA gene variants was observed in carriers of the genes FANCD2, FANCE, and FANCL in the HNSCC patient cohort relative to the 1000 genomes population.

For many FA or FA-related proteins upstream of the ID complex, their variants will, to some extent, impair the monoubiquitination of FANCD2 and FANCI and thus the tumor suppressive roles of FA signaling. For example, FANCL has many variants, such as the known substrate binding mutants (W212A, W214A and L248A, F252A, L254A, I265A), an FA mutation (R221C), and 14 cancer-associated mutations (F110S, I136V, L149V, L154S, A192G, E215Q, E217K, R221W, T224K, M247V, F252L, N270K, V287G, and E289Q) [89]. Given FANCL is a key component of the FA core complex responsible for the monoubiquitination of FANCD2 and FANCI, these FANCL variants would eventually affect the FA signaling. As all possible variants of many other FA or non-FA proteins, they too interfere with the function of the ID complex [90,91,92] and even the stability of the FANCD2 and FANCI proteins [93].

Our ongoing work indicates FA signaling is specifically involved in a novel aspect of cancer research and cancer health disparities [94]. In recognition of the biological factors that may enhance our understanding of the differences observed in cancer incidence, prevalence, morbidity, and mortality rates among different racial/ethnic minority populations [94,95], including Native Hawaiians (*Hawaii Cancer at a Glance*, 2012–2016), we decided to explore possible biological variations that are unique to Native Hawaiians with colorectal cancer (CRC). The RNA-seq on the 40 CRC tumor and matched normal samples (80 samples in total) were used to establish the first resource of The Cancer Genome Atlas (TCGA)-like datasets specifically for Native Hawaiians (NH) or Pacific Islanders (PI). Using this first-hand resource, we identified biological variations unique in NH/PI that impair FA signaling, which is not part of canonic causes known during colorectal oncogenesis. Now, we will follow this novel lead in order to uncover how FA signaling contributes to race-specific causes of CRC. As a consequence, our studies may promise valuable insights into CRC susceptibility and targets of intervention with the goal of positively impacting CRC health disparities, particularly those associated with NH/PI. 

### 2.4. Summary

**Figure 2:**
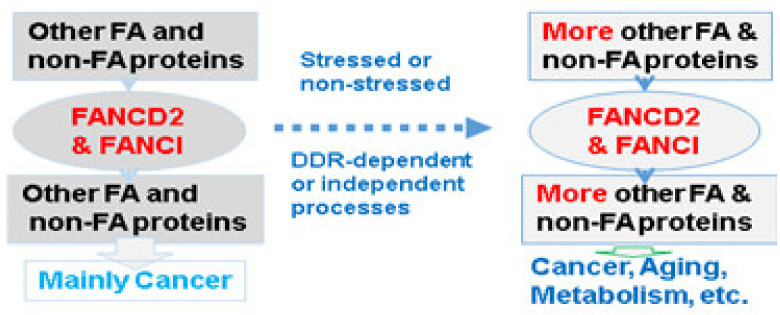
Illustrating functional roles of FA signaling with a relatively defined focal point.

A large body of research demonstrates that FA signaling is of extreme importance to facilitate an understanding of the pathogenesis of human diseases. Noting the indefinite nature of upstream and downstream FA signaling, its relatively defined center appears more capable of helping understand how FA signaling functions as a unique biological/genetic model system, which is a naturally existing advantage for biomedical researchers to take. We updated the functional varieties of FANCD2 and FANCI, the two players at the center of FA signaling. Both have well-known functioning upon DNA damage or during DNA replication, but also play important roles in the other phases of a cell cycle under both stressed and nonstressed conditions by participating in many biological processes, including RNA spacing, apoptosis, etc., aside from DDR. When FANCI or FANCD2 is not properly functioning, both can lead eventually not only to cancer as emphasized herein but also to aging and many other states, including metabolic disorders (Figure 2). 

Despite our emphasis on the functional roles of FANCD2 and FANCI in maintaining genome stability, it remains unclear how exactly FANCD2 and FANCI promote chromosomal stability when replication forks encounter abnormal DNA structures or tightly chromatin-bound proteins when probing the in-depth molecular insights into replication. Upon activation of the master replication checkpoint kinase ATR, FANCD2 and FANCI accumulate within chromatin in the vicinity of replication forks, and FANCD2 associates transiently with the replication machineries. It is also unclear as to the in-depth molecular insights into DDR in the maintenance of genome stability, which are attributed to FANCD2 and FANCI. FANCD2 may bind directly to histones and promotes the nucleosome assembly upon DNA damage or during DNA replication. Combined, these suggest that FANCD2 and FANCI regulate the further molecular machineries for chromatin-based processes and many others, preventing the human genome from going awry for cancer and other human diseases. 

With constant growth in its upstream and downstream, FA signaling emerges to be the biggest cellular defense-signaling network. However, we are able to appreciate its functional roles through the relatively defined axis, its focal point (FANCD2 and FANCI). FA signaling is involved in both DDR-dependent and independent cellular processes under stressed and nonstressed conditions. Consequently, FA signaling protects humans from a variety of diseases beyond cancer. 

## 3. Prospective

### 3.1. Is There an Evolutionary Path for FA Signaling to “Grow”?

**Figure 3:**
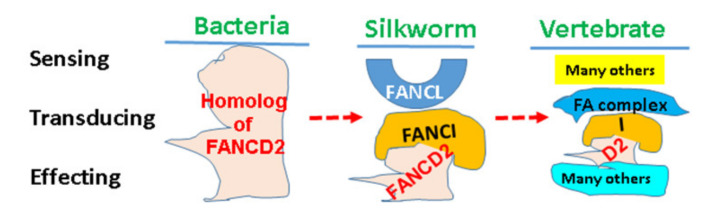
Schematic Outline of a Putative Evolutionary Path for the Focal Point of DDR/FA Signaling across Different Species. In bacteria, the homolog of FANCD2 may act as a sensor, transducer, and/or effector in response to genotoxic stresses. However, it receives a helper of FANCI and a regulator of FANCL in silkworm upon coupling with a variety of insults. FANCL, here, may act relatively more as a sensor. In vertebrate, FANCD2 and FANCI appear to be totally centered, which work in concert with many others to form a very complicated cellular defense signaling network.

Among FA genes, FANCD2 is the most conserved from bacteria to humans [96,97]. This differs from the other FA genes (with an exception of FANCM), many of which only exist in vertebrates and act in concert in response to DNA damage [98]. Silkworm, Bombyx mori (Bm), lacks apparent homologues of the FA core complex. However, BmFANCD2 and BmFANCI (the putative substrates of the FA complex) and BmFANCL (the putative catalytic E3 ubiquitin ligase of the FA complex) are conserved. Similarly, the monoubiquitination of silkworm FANCD2 depends on FANCI and FANCL, which are stabilized on chromatin after the treatment of DNA crosslinking agents. Therefore, “FA signaling” in B. mori apparently works in a similar manner as that in vertebrates but much simpler [99]. In eukaryotes, DDR/FA signaling is evidently becoming very complicated. As known, yeast cells require a combination of nucleotide excision repair, homologous recombination repair, and postreplication repair/translesion DNA synthesis to remove DNA crosslinks. In mammalian cells, the responses to DNA crosslinks require the coordination of complex signaling networks, which include the FA proteins and many non-FA proteins including ATM, ATR, and HHR6. However, it is barely known how mere FANCD2 functioning in bacteria is shaped into the biggest cell defense signaling network in mammalian cells (Figure 3).

### 3.2. Could an FA Gene or FA Signaling Be an Oncogenic Driver? 

In a study of molecular tumorigenesis of thymoma [100], the authors found that FANCI was one of genes upregulated up to 20-fold and formally considered as a driver oncogene. This is somehow consistent with our early awareness [82] and that of others [101], in which the extreme level of monoubiquitinated FANCD2 was considered oncogenic. This concept demands to be thoroughly explored. 

### 3.3. Is It a Better Therapeutic Strategy to Target the Focal Point of FA Signaling Than Any Others Functioning in DDR/FA Signaling? 

To date, numerous targets derived from either upstream or downstream of DDR/FA signaling are under testing to increase the sensitivity of chemotherapy [2,102,103,104]. Many of them may be promising initially; but nearly all would eventually develop resistance. In recognition of the most complicated cellular defense network of FA signaling developed in humans over any other species (Figure 1), targeting FANCD2 or FANCI would appear to be superior to others, which are not working at the focal point of DDR/FA signaling, to improve the sensitivity of chemotherapeutic agents.

## Data Availability

The work referenced is openly available in PubMed.

## Abbreviations

AML	acute myeloid leukemia
APS	alternative polyadenylation site
ATM	ataxia telangiectasia mutated
ATR	ATM and Rad3-related
BER	base excision repair
Bm	Bombyx mori
BMSC	bone marrow stromal cells
CRC	colorectal cancer
DDR	DNA damage response
FA	Fanconi Anemia
FAAP	FA-associated protein
FANCD2	FA D2 group protein, etc. similarly for other FA proteins
HHR6	*human homologs* of the yeast ubiquitin-conjugating enzyme Rad6
HNSCC	head and neck squamous cell carcinoma
HR	homologous recombination
ICL	interstrand crosslinks
IMPDH2	inosine monophosphate dehydrogenase 2
Me-D/-P	DNA methylation in the distal or proximal region
MMC	miotmycin C
NER	nucleotide excision repair
NHEJ	non-homologous end joining
NH/PI	Native Hawaiians or Pacific Islanders
PIDD1	P53-induced death domain protein 1
RAIDD	RIP-associated ICH1/CED3-homologous protein with a death domain
RPS27L	ribosomal protein S27-like
TCGA	The Cancer Genome Atlas
TFG	TRK-fused gene
TLS	translesion DNA synthesis
